# Defining the Minimal Important Difference in International Prostate Symptom Score for Men with Lower Urinary Tract Symptoms Using a Patient-centered Anchor Measure

**DOI:** 10.1016/j.euros.2025.09.003

**Published:** 2025-09-25

**Authors:** Laura Wiemer, Walter Lehmacher, Sandra Schönburg, Christian Gratzke, Kurt Miller, C. Patrick Papp

**Affiliations:** a Department of Urology, Charité – Universitätsmedizin Berlin, Berlin, Germany; b Kranus Health GmbH, Munich, Germany; c Institute of Medical Statistics and Computational Biology, University of Cologne, Cologne, Germany; d Department of Urology and Kidney Transplantation, Martin Luther University Halle Wittenberg, Halle (Saale), Germany; e BG Klinikum Bergmannstrost, Halle (Saale), Germany; f Department of Urology, University of Freiburg, Freiburg, Germany

**Keywords:** Lower urinary tract symptoms, International Prostate Symptom Score, Minimal important difference, Minimal clinically important difference, Benign prostatic enlargement, Randomized controlled trial, Digital health, Urology

## Abstract

**Background and objective:**

Few studies have investigated the minimal important difference (MID) for the International Prostate Symptom Score (IPSS), which is essential for interpreting clinical outcomes after treatment for lower urinary tract symptoms (LUTS). The aim of our study was to determine the IPSS MID using data from a randomized controlled trial (RCT) evaluating an app-based intervention (Kranus Lutera) for LUTS in men.

**Methods:**

Patients with LUTS diagnosed with benign prostatic hyperplasia, overactive bladder, or both by office-based urologists across Germany were included. After following the Kranus Lutera app for LUT therapy for 12 wk, participants completed the IPSS and the Patient Global Impression of Change (PGIC) questionnaire. The MID, defined as the mean change from baseline in IPSS among 82 men who reported “minimally improved” on the PGIC, was calculated in a secondary exploratory analysis.

**Key findings and limitations:**

The MID calculated for the IPSS was 5.26 points (95% confidence interval [CI] 4.38–6.13). Stratified analysis revealed a higher MID in the subgroup with severe baseline symptoms (8.23; 95% CI 6.58–9.88) than for the subgroup with moderate symptoms (4.00; 95% CI 3.14–4.86). As the data were drawn from a single RCT, the generalizability may be limited, particularly for results for subgroups with small sample sizes.

**Conclusions and clinical implications:**

These findings provide a valuable reference for clinicians interpreting treatment outcomes and offer important insights for future trial design. Further research in larger and more diverse populations is essential to improve generalizability and external validation.

**Patient summary:**

We looked at results for the International Prostate Symptom Score (IPSS), a questionnaire used to assess lower urinary tract symptoms (LUTS) in men, to determine what patients feel is a meaningful improvement in symptoms. We used data from a clinical trial that tested an app to help men in improving their LUTS. Men felt that the minimum level of improvement for a benefit was change of 5.26 points in IPSS. Men with more severe symptoms at the start of treatment required greater improvements, while men with moderate symptoms needed smaller changes. These findings may help doctors in understanding the amount of improvement in symptoms that patients feel is significant, and could guide future LUTS treatments and trials.

## Take Home Message

This analysis of data from the BEST trial provides a patient-centered estimate of a meaningful improvement in International Prostate Symptom Score. The findings suggest that a change of 5.26 points represents a noticeable benefit for patients. Men with more severe symptoms at baseline required a greater improvement than men with moderate symptoms. These results may offer valuable guidance for clinicians assessing treatment outcomes and important insights for future research.

## 1 Introduction

Lower urinary tract symptoms (LUTS) in men are highly prevalent and encompass a range of urinary complaints, including issues with storage, voiding, and postmicturition, that significantly impair quality of life and are associated with higher risk of cardiovascular events and mortality [[1], [2], [3]].

Given the impact of LUTS on daily life, early and accurate assessment is critical. International guidelines recommend structured symptom assessment using tools such as the International Prostate Symptom Score (IPSS) [4]. The IPSS is widely validated and has diagnostic and assessment roles in both clinical trials and routine care [5]. The IPSS is the patient-reported outcome measure (PROM) most frequently used in urology, covering approximately 60% of the global male population [6].

A key element in interpreting symptom scores in both research and clinical settings is the minimal important difference (MID), which is the smallest change in score perceived by patients as beneficial. An initial trial in 1995 comparing pharmacological therapies for benign prostatic hyperplasia (BPH) suggested a threshold of 3 points as the IPSS MID [7], while a more recent study in Dutch populations treated with α-blockers proposed 5.2 points [8]. Despite these efforts, IPSS MID estimates remain variable, with some studies instead defining a treatment response as an IPSS reduction of ≥25% [9,10].

Conservative and lifestyle-based interventions are widely recognized as effective first-line treatments for LUTS, either alone or as complements to pharmacological therapies [4,11]. However, adherence to such strategies can be challenging, particularly in the absence of structured support.

To address this problem, the BEST trial investigated app-based therapy that combines guideline-recommended physical, behavioral, and psychological components over 12 wk in 237 patients [12].

The intervention was based on a personalized digital therapy app (Kranus Lutera; Kranus Health, Munich, Germany) for treating LUTS that guides users through pelvic floor and bladder training, mental relaxation techniques, and educational content. The app features a micturition diary, adaptive exercises, urge control tools, and progress tracking to boost adherence and long-term improvement [12].

The aim of the present study was to use data from the BEST trial to calculate the MID for the IPSS in a broader context.

## 2 Patients and methods

BEST was a randomized, single-blind trial that assessed app-based therapy for LUTS [12]. The primary outcomes were treatment efficacy, while the secondary analysis described here was performed to estimate the MID for the IPSS using data from the trial. All participants were diagnosed with BPH-related LUTS or overactive bladder (OAB) by their treating physicians, with diagnoses subsequently confirmed by the study investigators. In addition, participants were required to have a symptom burden of IPSS ≥4 or of ≥18 points on the OAB Questionnaire Short Form (OAB-q SF) part 1. Exclusion criteria were conditions such as recurrent urinary retention, urinary tract infection, bladder stones, macrohematuria, structural abnormalities (eg, upper urinary tract dilation), and neurological disorders. Health state changes were evaluated using the Patient Global Impression of Change (PGIC) scale after therapy completion, which was at 12 wk for the intervention group and at 24 wk for the control group, as the control group had a waiting period of 12 wk before starting 12-wk therapy. The PGIC is a single-item measure whereby patients rate how their condition has changed since the start of treatment, and has been used in several previous studies [8,13]. The response options are presented on a 7-point Likert scale, ranging from “very much improved” to “very much worse”. MID was defined as the mean change in IPSS for participants reporting “minimally improved” on the PGIC. Only cases with complete data available were included in the analysis, so no imputation was performed. A Spearman correlation coefficient of ≥0.50 between IPSS changes and PGIC was considered ideal for producing reliable estimates of the MID [[14], [15], [16]]. Additional analyses were performed with stratification by symptom severity and International Classification of Disease (ICD) diagnosis codes on the assumption that patients with more severe baseline symptoms may require a greater absolute change to perceive a meaningful improvement. The IPSS categorizes symptom severity as mild (1–7 points), moderate (8–19 points), and severe (20–35 points).

Statistical analysis was conducted using SAS (v9.4), R (v4.4.2), and RStudio (v2024.09.1). The data set used for MID calculations contained only the patients who provided IPSS and PGIC scores at follow-up.

## 3 Results

Of the 237 participants randomized in BEST, 172 responded to the PGIC questionnaire. At baseline, mean IPSS was 17.65 points in both the intervention group and the control group. Approximately 60% of the patients had moderate IPSS, while approximately one-third had severe symptoms. Around 30% were taking some form of LUTS medication, and ∼20% had received additional LUTS therapies (Supplementary Table 1).

The Spearman correlation coefficient between the IPSS change from baseline and the PGIC score was exactly 0.50 (*p* < 0.0001), meeting the targeted threshold.

Among the 172 patients who provided both IPSS and PGIC data, 82 rated the change after app therapy as “minimally improved” on the PGIC. This subgroup reported a mean IPSS change of 5.26 points from baseline.

Supplementary Table 2 shows the change in IPSS for each of the seven PGIC response options.

**Fig. 1:**
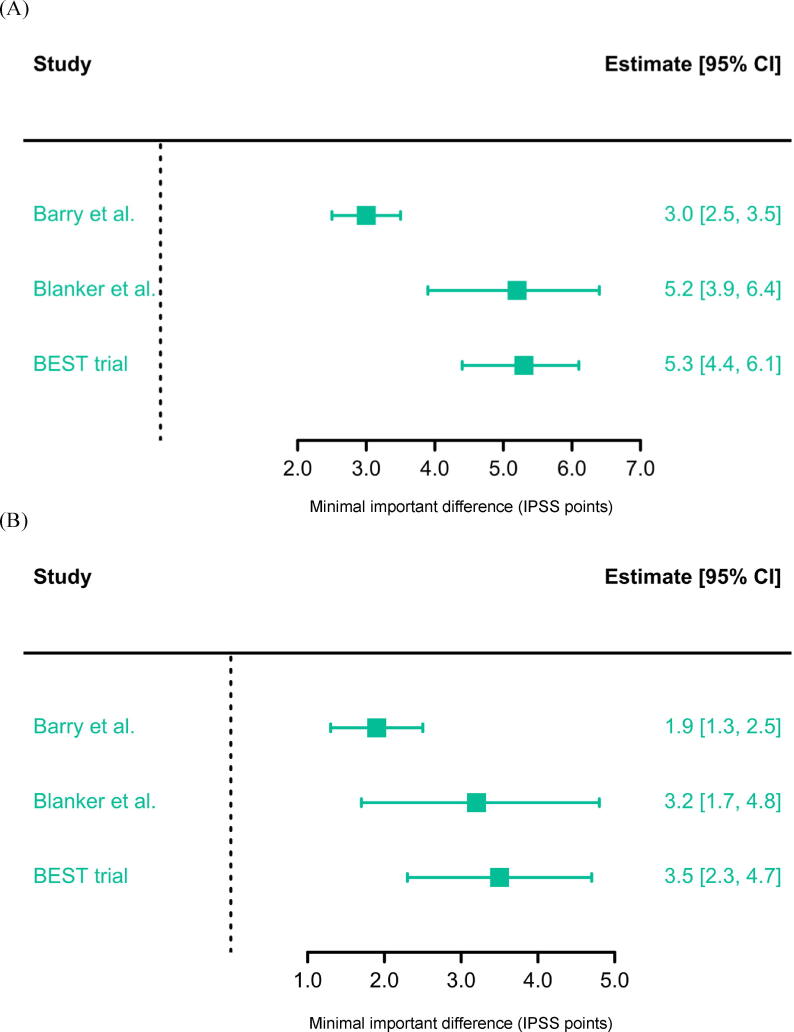
Forest plots of IPSS MID estimates for (A) patients with symptoms of any severity and (B) the subgroup with moderate IPSS severity at baseline according to three different studies (Barry et al. [7], Blanker et al. [8], and the BEST trial [12]). Squares denote the MID point estimates; horizontal lines show the 95% confidence interval, indicating the range of plausible MID values. The plots highlight the variability in MID estimates across different study populations and methodologies, but general consistency in magnitude, with all values falling between 3.0 and 5.3 IPSS points for patients with symptoms of any severity at baseline. MID values tend to be lower for patients with moderate symptoms (in B) than for those with more severe symptoms (not shown, patients with symptons of any severity shown in A). CI = confidence interval; MID = minimal important difference; IPSS = International Prostate Symptom Score.

The IPSS MID of 5.26 points derived from the BEST cohort aligns closely with the 5.2-point estimate by Blanker et al. [8] but exceeds the 3-point threshold reported by Barry et al. [7] (Fig. 1A) [7].

### 3.1 Subgroup analyses

While medication status had no effect on the IPSS MID, stratification by baseline IPSS severity revealed that the increased MID with symptom severity: 1.67 for mild (*n* = 3), 4.00 for moderate (*n* = 53), and 8.23 for severe symptoms (*n* = 26; Table 1).

**Table 1:** Subgroup analyses for patients reporting “minimally improved” on the Patient Global Impression of Change questionnaire

Subgroup of the “minimally improved” cohort	*N*	Mean IPSS at	Change in IPSS for “minimally improved” cohort	
		baseline (SD)	Mean change (95% CI)	SD
**LUTS medication use**				
No LUTS medication	56	16.86 (6.69)	−5.23 (−6.15 to −4.31)	3.44
LUTS medication	26	18.19 (5.89)	−5.31 (−7.33 to −3.29)	5.00
**IPSS severity**				
Mild	3	8.67 (3.79)	−1.67 (−3.11 to −0.23)	0.58
Moderate	53	14.15 (3.63)	−4.00 (−4.86 to −3.14)	3.11
Severe	26	24.65 (4.42)	−8.23 (−9.88 to −6.58)	4.09
**ICD code**				
ICD N40 (BPH) alone	41	16.61 (5.73)	−4.63 (−5.99 to −3.27)	4.31
ICD N32.8 (OAB) alone	23	16.87 (7.75)	−5.61 (−7.21 to −4.01)	3.69
ICD N40 and N32.8	18	19.33 (6.06)	−6.22 (−7.92 to −4.52)	3.42
**IPSS severity for ICD N40**				
Moderate	30	14.10 (3.92)	−3.50 (−4.72 to −2.28)	3.28
Severe	11	23.45 (4.03)	−7.73 (−11.30 to −4.13)	5.35

BPH = benign prostatic hyperplasia; ICD = International Classification of Diseases; IPSS = International Prostate Symptom Score; LUTS = lower urinary tract symptoms; OAB = overactive bladder; SD = standard deviation.

In terms of diagnoses, the IPSS MID was 4.63 points in the subgroup with BPH, and was higher in the subgroup with OAB, at 5.61 points. Patients with both conditions had the highest IPSS MID of 6.22 points. When considering only patients with an isolated BPH diagnosis and stratifying by severity, the IPSS MID estimate was 3.50 points (*n* = 30) for the moderate subgroup and 7.73 points (*n* = 11) for the severe subgroup (Table 1).

Fig. 1B shows a forest plot of IPSS MID values calculated for the subgroup with BPH and symptoms of moderate severity across various studies, which range from 1.9 to 3.5.

## 4 Discussion

The BEST trial evaluated an app-based intervention involving conservative therapies recommended by LUTS-specific guidelines [12]. The digital intervention demonstrated improvements in symptom burden and quality of life comparable to those with pharmacotherapy [12,17]. The PGIC scale used in the trial allowed anchor-based calculation of the IPSS MID. This approach provides additional patient-centered evidence for estimation of the true clinical relevance of an intervention measured via the IPSS questionnaire.

According to data from the BEST trial and the PGIC anchor, an IPSS change of 5.26 points represents the smallest improvement perceived as meaningful by patients. This estimate is consistent with a Dutch study that reported an IPSS MID of 5.2 points [8], although with narrower confidence intervals, and notably higher than the 3-point estimate proposed by Barry et al. [7].

While some studies defined a response as an IPSS reduction of ≥25% [9,10], our anchor-based IPSS MID estimate of 5.26 points corresponds to an average 30% reduction from baseline and align with the German Institute for Quality and Efficiency in Health Care recommendation of a ≥15% reduction in total score as a benchmark for a patient-relevant benefit [18,19], which corresponds to 5.25 points. This suggests that the 25% threshold may not fully capture the degree of change patients perceive as meaningful, particularly for those with a higher symptom burden at baseline. Anchor-based methods, which reflect patients’ evaluation of improvement, are considered more clinically relevant and should be preferred when available. Percentage-based thresholds may be useful in the absence of anchor data, but risk misrepresenting patient-perceived benefits.

The exploratory subgroup analyses revealed that IPSS MID values varied by baseline severity and diagnosis. Because patients with higher IPSS at baseline have greater room for an absolute improvement, they may yield larger effect sizes than those with milder symptoms. Conversely, those starting with a low symptom level at baseline cannot achieve large absolute gains. We therefore estimated IPSS MIDs stratified by baseline severity, in line with previous studies [8]. Nonetheless, this severity‐dependent approach may still bias comparisons across groups. The small number of participants with mild symptoms at baseline limits the reliability of the IPSS MID estimate for this subgroup, and any interpretation should be made with caution. Thus, this subgroup was not the focus of our main analysis. However, the IPSS MID values for participants with moderate or severe symptoms were based on larger sample sizes and showed that higher baseline severity was associated with greater MID values, consistent with the literature [8]. In addition, patients with dual diagnoses (BPH and OAB) had slightly higher IPSS MID values, suggesting that perceived improvements may be influenced by the underlying pathophysiology. The higher MID in the combined diagnosis group may reflect the slightly elevated IPSS at baseline and the need for greater improvement to achieve clinical meaningfulness. While data on prostate volume were not systematically collected for the purpose of this MID analysis, treating physicians who diagnosed and enrolled patients were free to use prostate imaging or other diagnostics as part of their routine assessment. Thus, our IPSS MID estimates reflect real-world symptom-driven treatment decisions, with a focus on PROMs rather than anatomic or urodynamic measures.

MID values can vary by treatment type, patient population, disease severity, and estimation method (eg, anchor- vs distribution-based) and should be interpreted within their specific clinical and methodological context [20,21]. Notably, our results align closely with prior studies on pharmacological treatments (α-blockers), and subgroup analysis showed that concomitant LUTS medication had no impact on IPSS MID values, suggesting robustness and potential generalizability across conservative and pharmacological therapies. However, caution is advised when applying these values to invasive interventions, for which patient expectations and symptom relief may differ. The study benefits from a prospective design and rigorous assessment, but small sample sizes for certain subgroups limits the precision of the estimates. While the confidence intervals are narrower than those presented by Blanker et al. [8], a greater sample size would further enhance the precision. The study population was drawn from a single country and one randomized controlled trial, which may limit the generalizability. Further research across different populations, health care systems, and intervention types is essential to improve the generalizability and external validation.

It is important to consider whether the digital delivery format may have influenced patients’ perception of improvement. Importantly, PROMs such as the IPSS and PGIC were collected via clinical data management software combined with structured telephone interviews conducted by an independent clinical research organization, rather than directly via the app. This method of data collection adds separation between the intervention and outcome assessment, which reduces the influence of expectancy effects and self-reporting bias. While all PROMs are inherently subjective, this approach improves the internal validity of the findings by reducing the risk of inflated symptom improvement due to the digital format alone.

The estimates in this study are higher than those reported by Barry et al. [7], potentially because of variations in follow-up duration, symptom severity, and global assessment tools. Use of a 7-point Likert scale here and in the study by Blanker et al. [8] may have contributed to variations. In addition, the inclusion of men with severe symptoms and those on medication may explain the greater changes observed.

The IPSS is a widely used PROM and is recommended by LUTS guidelines, with MID estimates ranging from 3 to 5 points. These variations highlight the potential impact of baseline symptom severity, population characteristics, and methodological differences on determining the threshold for clinical relevance.

## 5 Conclusions

Our study provides a patient-centered MID estimate for the IPSS of 5.26 points. This value may aid in clinical interpretation of results, especially for nonpharmacological treatments. Limitations in sample size and generalizability should be considered when applying these estimates more broadly.

  ***Author contributions***: Laura Wiemer had full access to all the data in the study and takes responsibility for the integrity of the data and the accuracy of the data analysis.

  *Study concept and design*: Lehmacher, Papp, Wiemer.

*Acquisition of data*: Schönburg, Gratzke.

*Analysis and interpretation of data*: Lehmacher, Papp, Wiemer.

*Drafting of the manuscript*: Papp, Wiemer.

*Critical revision of the manuscript for important intellectual content*: Miller, Gratzke, Schönburg, Lehmacher.

*Statistical analysis*: Papp, Wiemer.

*Obtaining funding*: None.

*Administrative, technical, or material support*: Lehmacher.

*Supervision*: None.

*Other*: None.

  ***Financial disclosures:*** Laura Wiemer certifies that all conflicts of interest, including specific financial interests and relationships and affiliations relevant to the subject matter or materials discussed in the manuscript (eg, employment/affiliation, grants or funding, consultancies, honoraria, stock ownership or options, expert testimony, royalties, or patents filed, received, or pending), are the following: C. Patrick Papp, Kurt Miller, and Laura Wiemer report employment with Kranus Health. The remaining authors have nothing to disclose.

  ***Funding/Support and role of the sponsor*:** The primary trial was supported by Kranus Health, but the secondary analysis presented here received no funding.
