# Supplementary material for: Defining the Minimal Important Difference in International Prostate Symptom Score for Men with Lower Urinary Tract Symptoms Using a Patient-centered Anchor Measure
Source: Eur Urol Open Sci. 2025 Sep 25;81:44–9. doi: 10.1016/j.euros.2025.09.003 (PMC12509103; doi:10.1016/j.euros.2025.09.003)
Supplement: Supplementary Data 1 [file mmc1.docx]

# Supplementary Tables

Table S 1. Summary of Measurements: Absolute Values with Percentages

| Variable | Completed Intervention | Completed Control | Dropout Intervention | Dropout Control |
| --- | --- | --- | --- | --- |
| n | 106 | 124 | 6 | 1 |
| Age Mean | 58.41 | 57.98 | 63.33 | 79 |
| Age SD | 12.07 | 12.58 | 8.07 | - |
| Referred by GP | 1 (0.94%) | 2 (1.61%) | 0 (0.00%) | 0 (0.00%) |
| Referred by Urologist | 87 (82.08%) | 101 (81.45%) | 6 (100.00%) | 1 (100.00%) |
| Other | 18 (16.98%) | 21 (16.94%) | 0 (0.00%) | 0 (0.00%) |
| ICD N40 | 50 (47.17%) | 56 (45.16%) | 4 (66.66%) | 0 (0.00%) |
| ICD N32.8 | 31 (29.25%) | 42 (33.87%) | 1 (16.67%) | 0 (0.00%) |
| Both ICDs | 25 (23.58%) | 26 (20.97%) | 1 (16.67%) | 1 (100.00%) |
| IPSS Mean | 17.65 | 17.65 | 13.67 | 24.00 |
| IPSS SD | 6.17 | 6.02 | 4.97 | - |
| IPSS Category Mild | 6 (5.66%) | 4 (3.23%) | 0 (0.00%) | 0 (0.00%) |
| IPSS Category Moderate | 63 (59.43%) | 71 (57.26%) | 5 (83.33%) | 0 (0.00%) |
| IPSS Category Severe | 37 (34.91%) | 49 (39.52%) | 1 (16.67%) | 1 (100.00%) |
| Current medication for bladder voiding dysfunction | 31 (29.25%) | 38 (30.65%) | 0 (0.00%) | 0 (0.00%) |
| Previous therapies for bladder voiding dysfunction | 23 (21.70%) | 27 (21.78%) | 0 (0.00%) | 0 (0.00%) |
| Previous surgery on the prostate | 1 (0.94%) | 8 (6.45%) | 0 (0.00%) | 0 (0.00%) |

Table S 2. IPSS Mean Change from Baseline for each PGIC category

| PGIC Category | N | Mean Change (IPSS points) | SD | 95% CI | |
| --- | --- | --- | --- | --- | --- |
| 1 - Very Much Improved | 10 | -10.40 | 5.93 | -14.60 | -6.16 |
| 2 - Much Improved | 57 | -9.16 | 5.57 | -10.60 | -7.68 |
| 3 - Minimally Improved | 82 | -5.26 | 3.97 | -6.13 | -4.38 |
| 4 - No Change | 19 | -2.58 | 2.95 | -4.00 | -1.16 |
| 5 - Minimally Worse | 4 | -1.00 | 1.83 | -1.91 | 3.91 |
| 6 - Much Worse | 0 | - | - | - | - |
| 7 - Very Much Worse | 0 | - | - | - | - |
